# Management of adult acute lymphoblastic leukemia in the Gulf Cooperation Council (GCC) countries: A consensus report from the GCC Adult ALL Working Group

**DOI:** 10.1002/cnr2.1931

**Published:** 2023-12-11

**Authors:** Feras Alfraih, Ahmed Absi, Mohamed Abuhaleeqa, Khalofa Alghamdi, Ahmad Alhuraiji, Murtadha Al‐Khabori, Zeyad Al‐Shaibani, Musa Alzahrani, Honar Cherif, Saleem Eldadah, Amr Hanbali, Ibraheem H. Motabi, Hind Salama

**Affiliations:** ^1^ King Faisal Specialist Hospital and Research Centre Riyadh Saudi Arabia; ^2^ Al Faisal University Riyadh Saudi Arabia; ^3^ Princess Noorah Oncology Center King Abdulaziz Medical City, Ministry of National Guard Health Affairs Jeddah Saudi Arabia; ^4^ Abu Dhabi Stem Cells Center Abu Dhabi UAE; ^5^ King Abdullah Medical City Makkah Saudi Arabia; ^6^ Department of Hematology Kuwait Cancer Control Center Kuwait City Kuwait; ^7^ Department of Hematology, College of Medicine and Health Sciences Sultan Qaboos University Muscat Oman; ^8^ King Faisal Specialist Hospital and Research Centre Madinah Saudi Arabia; ^9^ Department of Medicine, College of Medicine King Saud University Riyadh Saudi Arabia; ^10^ National Center for Cancer Care and Research Hamad Medical Cooperation Doha Qatar; ^11^ King Fahad Medical City Department of Adult Hematology and BMT; ^12^ King Abdulaziz‐Medical City Riyadh Saudi Arabia

**Keywords:** acute lymphoblastic leukemia, adult, chemotherapy, consensus, standard of care

## Abstract

Leukemia burden is growing in the Gulf Council Cooperation (GCC) countries. Nonetheless, there is no unified protocol for managing adult acute lymphoblastic leukemia (ALL) patients in the GCC‐countries. Therefore, the GCC Adult‐ALL Treaters working group developed this consensus to address the adult‐ALL treatment protocols in the GCC‐countries and related toxicities' management. Besides, the consensus aimed to highlight the current unmet needs and treatment gaps and provide recommendations to optimize adult‐ALL care and patient‐centered communication. A three‐step modified Delphi method to develop evidence‐based recommendations through two‐voting rounds and in‐between virtual meetings are used in the manuscript development. A 12 experts' panel from five GCC‐countries and two international experts were invited to participate in this consensus. This consensus consisted of 35‐statements that highlighted the experts' recommendations to optimize ALL adults' care in the first line setting and manage pediatric or pediatric‐inspired regimens‐related toxicities. Besides, guidance was provided for future research direction and improve patient‐centered communication. In conclusion, the adult‐ALL management landscape is evolving, and the current evidence highlights better response and survival outcomes with pediatric or pediatric‐inspired regiments. Therefore, protocols are needed to optimize the adult‐ALL management in the GCC and tailored clinical‐trials findings according to the GCC patients' characteristics and local‐healthcare infrastructure.

## 1 INTRODUCTION

Acute lymphoblastic leukemia (ALL) is a hematological malignancy affecting both children and adults, with a peak incidence between 1 and 4 years. The condition develops due to a malignant proliferation of immature lymphoid cells, with a secondary invasion of the blood, bone marrow, and extramedullary sites.^1^ According to recent epidemiological figures, the global incidence rate of ALL notably increased by 30.81% over the period between 1990 and 2017 to reach 64 190 new cases in 2017, accounting for nearly 12% of the global leukemia cases.^2^ In 2022, the American Cancer Society estimated 6660 new cases and 1560‐related deaths in the United States.^3^ The incidence of ALL shows a slight male‐predominance and an age‐specific trend, being highest in childhood and adolescence (nearly 60% of the cases) and lowest among patients aged 25–45 years old.^4^

Several risk factors are described in the development of ALL, including genetic susceptibility and chromosomal alterations, infection, and ionizing radiation.^1^

With recent medical advances and enhanced understanding of the underlying molecular mechanisms in ALL, in addition to risk stratification, next‐generation sequencing, and targeted therapies, the outcomes of ALL have shown significant improvements, particularly in children age group, with an increased 5‐year overall survival (OS) of ALL from 25% at the late 1970s to over 90% by 2010, which was consistent across low, middle, and high‐income countries^5^ However, the adult group of ALL population still present suboptimal long‐term survival despite the considerable improvement in diagnostic and therapeutic approaches. Recent statistics showed that the 5‐year OS in adult ALL patients is 60%–70% in younger adults, compared to 89% in patients under 20 years old.^6^ Adult patients—especially those older than 55–65 years old—show a high proportion of Ph/BCR‐ABL positive ALL and open present poorer performance status and multiple comorbidities, which limits their ability to receive aggressive chemotherapy.^7^ Immunophenotyping, fluorescent in situ hybridization (FISH) analysis, molecular diagnostics, establishing assays for MRD testing, and other risk stratification tools are crucial in management decisions and appropriateness to standard protocols in adult ALL patients. Pediatric‐based protocols such as the German Multicenter Study Group for Adult Acute Lymphoblastic Leukemia (GMALL) are used in many countries as a first‐line option for adult ALL patients, while the Hyper‐CVAD protocol is used in other countries.^8^, ^9^ The latest version of the National Comprehensive Cancer Network stated that fit adult ALL patients can receive intensive backbone regimen of vincristine, a corticosteroid, asparaginase and an anthracycline for induction, followed by consolidation cycles with high‐dose methotrexate, cytarabine, asparaginase and several other compounds including a reinduction and maintenance therapy, as well as allogeneic stem cell transplantation (SCT) in first remission for high‐risk patients.^8^ Patients with central nervous system (CNS) involvement are usually treated with the same protocols with or without radiotherapy and additional intrathecal therapies.^9^ Tyrosine kinase inhibitors (TKIs) exhibited promising results in Ph‐positive ALL with different backbones and stem cell transplantation, with a 2‐year remission rate of 81%.^10^ Pediatric‐inspired protocols have emerged as feasible and effective regimens for adolescents and young adults (AYAs) ALL patients and significantly improved long‐term survival.^11^, ^12^ Nonetheless, several treatment gaps and unmet needs are still present in the management landscape of adult ALL patients, including suboptimal OS, a lack of unified diagnostic and prognostic algorithms, and a high rate of treatment‐related toxicities.^13^ Therefore, efforts have been established to optimize the management of adult ALL patients, such as the GMALL.^14^

In the Gulf Council Cooperation (GCC) the leukemia burden is growing. According to the Saudi Cancer Registry, leukemia is the fifth most common cancer in Saudi Arabia, with ALL accounting for nearly one‐third of the cases.^15^ Besides, epidemiological figures showed a steady increase in leukemia incidence in Saudi Arabia over 15 years (from 1999 to 2013).^16^ Similarly, it was reported that the incidence of adult ALL in the United Arab Emirates was comparatively higher than in Western countries.^17^ The situation is not different in Kuwait, leukemia is the fifth leading cancer type in Kuwait according to the most recent health statistics with an estimated incidence of 1.1 per 100 000 persons in 2020.^18^, ^19^ Despite the growing burden of ALL in the GCC countries, there is no unified protocol for the management of adult ALL countries despite the trend of increased ALL cases in the region. Pediatric international protocols are well established and implemented, however, the limitations in ALL adult management and specificities of GCC patients urge the development of regional treatment protocol. Therefore, the GCC Adult ALL Treaters working group developed this consensus to address the treatment protocols for adult ALL in the GCC countries and management of related toxicities, highlight the current unmet needs and treatment gaps, and provide a set of recommendations to optimize adult ALL care and patient‐centered communication.

## 2 MATERIALS AND METHODS

### 2.1 Consensus design and panel recruitment

We adopted a three‐step modified Delphi method to recruit a panel of 12 experts from five GCC countries; all experts were consultant hematologists and affiliated with academic institutions from Saudi Arabia (*n* = 8), United Arab Emirates (*n* = 1), Qatar (*n* = 1), Oman (*n* = 1), and Kuwait (*n* = 1). A non‐probability convenient selection process was employed to recruit the experts, ensuring a geographical representation of major academic institutions in GCC. The Saudi Hematology Society invited the experts via email to participate in the working group and develop the present consensus. Besides, two international experts were invited to participate in the virtual meeting to present the experience of optimizing adult ALL management in Germany and the United States. All panel members were required to sign a disclosure statement before consensus development. This Delphi‐based consensus consisted of two voting rounds and an in‐between virtual meeting.

### 2.2 Literature review and statements development

The Saudi Hematology Society allocated a survey development committee to conduct a comprehensive literature search on Medline via PubMed to retrieve relevant guidelines, consensus, and systematic reviews about the management of adult ALL patients and unmet treatment gaps. The literature search was performed using the following MeSH terms: ((acute lymphoblastic leukemia [MeSH Terms]) AND (adult [MeSH Terms])) AND (care standard [MeSH Terms]). There was no language, year of publications, or country‐specific restrictions on the literature search. Data were retrieved only from level 1 quality of evidence, as classified by Wright et al.^20^ According to the search results, a framework was developed and contained the following domains: optimizing care for adults with ALL in the first‐line setting; management of toxicities for adults with ALL in the first‐line setting; and recommendations for developing management protocols and improving patient‐centered communication. A set of statements were developed for each domain.

### 2.3 Delphi process and consensus development

Experts were asked to vote on the first set of statements by the survey development committee. All statements were formulated as binary questions (agree/disagree), while the experts had the option to abstain from voting on each statement. A consensus was defined as an agreement level ≥ 75%.^21^ A virtual meeting was held to present the voting results and gather experts' insights on statements that did not reach the consensus level. Accordingly, the second round of votes was held, and the revised statements were sent out to the experts. Besides, the experts provided their views and recommendations for the educational strategy needed to optimize patient‐centered communication and delivery of care; these views and recommendations were summarized in this manuscript. The final consensus statements and manuscript were reviewed and approved by all panel members.

## 3 RESULTS AND DISCUSSION

Forty‐two statements were emailed to the experts for the first voting round. Of them, 11 statements reached the consensus level, and the remaining statements were discussed during the virtual meeting. The panel agreed during the meeting to remove six statements, add one new statement, and rephrase 25 statements for the second round of voting. Out of the 25 statements, two statements did not reach the consensus level. Thus, the present consensus was composed of 35 statements.

### 3.1 Optimizing 1st line management of adult ALL patients

In the present consensus, 11 recommendations were developed regarding optimizing the case of adult ALL patients in the first‐line setting (Table 1).

**TABLE 1:** GCC experts' consensus statements on optimizing 1st line management of adult ALL patients.

No.	Statement	%
1	Karyotyping analysis, fluorescent in situ hybridization (FISH) analysis, remains an essential component of the workup of newly diagnosed ALL in adults to identify Philadelphia chromosome status. In addition to distinguishing B‐cell ALL from T‐cell ALL, a detailed phenotypic analysis is essential at diagnosis to determine whether targeted antibody‐ based therapies may be appropriate.	100
2	A combination of flow cytometry and specialized FISH panels can be useful in the characteristic gene fusions and alterations.	100
3	Evaluation of the central nervous system with diagnostic lumbar puncture, including cytologic analysis of the CSF, should be routinely performed in patients with ALL	91
4	Most pediatric treatment protocols are based upon regimens consisting of 6 to 9 months of intensive induction/ consolidation/delayed intensification therapy, followed by prolonged maintenance and relying on less myelosuppressive chemotherapy.	100
5	Accumulating data increasingly supports treating AYAs with ALL with a pediatric and pediatric‐inspired regimen, considering it a standard of care for newly diagnosed younger adults with Ph– ALL.	100
6	Pediatric‐inspired regimens are preferred for young adults with T‐cell ALL.	91
7	Pediatric inspired regimens and regimens not incorporating asparaginase, may be recommended for T‐ALL in young adults	100
8	Dose‐adjusted asparaginase (including lowering and/or capping doses) in adults is generally tolerable in patients with ALL across age spectrums. In addition, pediatric inspired regimens may offer other benefits, such as fewer late occurring health complications, such as retaining fertility, which reflects on the long‐term cost to the health care system	100
9	Adherence to the prolonged maintenance course as the final phase of treatment in pediatric inspired regimens is crucial since it typically lasts 2 to 3 years in duration	82
10	Assessment of MRD response following frontline therapy is recommended in patients with ALL as an important prognostic feature, regardless of patient age or the frontline regimen used.	100
11	Available MRD evaluation methods are recommended, including multiparameter flow cytometry and quantitative polymerase chain reaction. However, detection of ALL MRD evaluating clonal immunoglobulin heavy chain and/or TCR gene rearrangements provides higher analytic sensitivity and a lower false‐negative rate.	100

Abbreviations: ALL, acute lymphoblastic leukemia; AYAs, adolescent and young adults; CSF, cerebrospinal fluid; FISH, fluorescent in situ hybridization; MRD, measurable residual disease; TCR, T‐cell receptor.

Alongside bone marrow aspiration and biopsy, karyotyping and fluorescent in situ hybridization (FISH) analysis are essential parts of the diagnostic workup of adult ALL. Nearly 75% of the ALL patients have abnormal karyotyping; of them, the Philadelphia chromosome (Ph), also known as e t(9;22) (q34;q11), accounts for 20%–30% of the abnormalities, with a steady increase in its frequency with age.^22^ Before introducing TKIs, Ph‐positive ALL was historically associated with adverse survival outcomes and represents poor prognostic factors. However, the landscape of ALL is evolving and other B‐subtypes have become the leading negative prognostic factor for ALL, alongside KMT2A gene rearrangements, del(17p), and complex abnormalities.^23^, ^24^ Thus, the panel agreed that karyotyping and FISH analysis should be an integral part of the diagnostic workup of adult ALL patients owing to the prognostic significance of Ph and other chromosomal abnormalities (s*tatement 1*).

Besides, multicolor flow cytometry (MFC) should be requested at diagnosis to distinguish between B and T cells lineage ALL and to identify sensitivity to targeted antibodies.^25^ For example, the introduction of CD20 monoclonal antibodies significantly improved the survival of B cell ALL with CD20 expression.^26^, ^27^ At the same time, intensive pediatric regimens with asparaginase prolonged the survival of early T cell precursor (ETP) ALL.^28^ The panel identified phenotypic analysis as an essential step in the diagnostic workup to determine the potential benefits of targeted antibodies (*statement 1*). They also agreed that combing MFC and FISH analysis can further benefit in identifying gene fusions and alterations (*statement 2*).

Since CNS involvement is present in nearly 5%–10% of adult patients with ALL at diagnosis and confers a poor prognosis, prophylactic treatment for possible occult CNS disease is recommended; nonetheless, the effectiveness of such prophylactic measures has been challenged in some reports.^9^ The experts agreed that diagnostic panel for CNS involvement should be routinely performed at diagnosis and involve lumbar puncture and cytologic analysis of the cerebrospinal fluid (CSF; *statement 3*). In adults, CNS leukemia is usually diagnosed based on the presence of ≥5 leukocytes/mL or cranial nerve palsies; besides, elevated protein levels may be present in the CSF of affected patients.^9^

Pediatric‐based regimens such as the GMALL protocols were suggested for the first‐line management of adult ALL patients under 65 years in many centers, while the Hyper‐CVAD protocol, which does not contain asparaginase, has been validated as an effective and tolerable alternative.^29^, ^30^ The latest version of the National Comprehensive Cancer Network stated that fit adult ALL patients receive an intensive backbone regimen of vincristine, a corticosteroid, and an anthracycline, as well as allogeneic stem cell transplantation (SCT) in the first remission.^8^ The intensity of the chemotherapeutic approaches depends mainly on the age and performance status, with younger patients usually receiving higher cumulative doses of corticosteroids, vincristine, and asparaginase than elderly patients.^7^ Despite the recent advances, suboptimal survival in adult ALL patients is still observed, which is attributed to the high rate of poor prognostic features, low rate of favorable features, and high treatment‐related toxicities, especially with more intensive regimens.^22^ On the other hand, the standard pediatric treatment protocols usually consist of 6–9 months of intensive induction and consolidation, followed by prolonged maintenance (*statement 4*). These pediatric regimens are composed of more intensive asparaginase and corticosteroids, which can lead to a higher rate of toxicities in frail older adults.

A growing body of evidence supports the survival benefits of pediatric and pediatric‐inspired regimens in AYAs patients with ALL (*statement 5*). Several retrospective studies demonstrated that AYAs patients exhibited better long‐term survival after receiving intensive pediatric protocol than historical cohorts who did not.^31^, ^32^ This was then followed by several prospective studies showing a 5‐year OS between 60% and 70% in AYAs patients receiving pediatric‐inspired intensive protocols.^11^, ^33^, ^34^ The preliminary results of GMALL Trial 08/2013 showed promising outcomes of the pediatric‐based therapy with more intensified peg‐asparaginase in newly‐diagnosed adults with ALL; in the AYAs subgroup, the 3‐year OS was 74%–87%.^35^ In another recent single‐arm study of ALL patients up to 60 years old, pediatric‐based therapy with peg‐asparaginase resulted in a complete response of 97% and a 3‐year OS of 76.4%.^36^ Based on these results, the panel agreed that pediatric‐inspired regimens and regimens without asparaginase are preferred for AYAs with T‐cell ALL (*statements 6 and 7*). The pediatric‐specific regimens can also reduce long‐term healthcare expenditure by incurring fewer complications, such as infertility (*statement 8*). However, the experts stated that the benefits of pediatric‐inspired regimens depend primarily on the adherence to the maintenance phase, which can last for 2–3 years (*statement 9*).

Although most adult ALL patients achieve complete remission (CR) on first‐line therapy, the relapse rate is still high, approaching nearly 40%, which has been linked to the presence of resistant measurable residual disease (MRD) undetectable with the standard cytomorphological assessment. MRD assessment using MFC has been validated as a reliable prognostic tool for ALL^37^; in adults, event‐free survival for patients with MRD negativity was 64% versus 21% in MRD‐positive patients.^38^ Current guidelines recommend MRD assessment after the first cycle of induction, at the achievement of CR, and then every 3–6 months. The presence of MRD plays a role in deciding subsequent treatment of the patients with allogeneic stem cell transplant or blinatumomab.^22^ However, MRD evaluation is complex and requires well‐equipped laboratories. The panel agreed that MRD assessment is recommended to assess the response to first‐line treatment in all age groups of ALL patients owing to its prognostic significance (*statement 10*). Thus, centers in GCC countries should ensure the availability of essential MRD evaluation workups, including MFC. However, the availability of tools assessing clonal immunoglobulin heavy chain and/or TCR gene rearrangements can provide a higher diagnostic yield (*statement 11*).

### 3.2 Management of toxicities during 1st line management of adult ALL patients

Despite the recent advances in novel therapies, such as monoclonal antibodies and TKIs, cytotoxic chemotherapy remains the recommended frontline regimen for adult ALL, with a high burden of associated toxicities.^39^ In this consensus, 17 statements were developed for managing toxicities during the frontline regimen for adult ALL (Table 2).

**TABLE 2:** GCC experts' consensus statements on management of toxicities during 1st line management of adult ALL patients.

No.	Statement	%
12	Toxicity profiles differ across different regimens. Asparaginase‐related toxicity, including hepatotoxicity, pancreatitis, and thrombosis, is greater in the pediatric‐inspired regimen. However, greater myelosuppression‐associated complications and grade 3 infections are related to hyper‐CVAD approach.	100
13	Toxicities associated with dose‐ adjusted peg‐asparaginase are manageable in most adult patients, and fatal outcomes from peg‐asparaginase toxicities are rare.	100
14	Understanding the mechanisms of peg‐asparaginase toxicities and patient‐associated risk factors is crucial to prepare adequate preventive measures and early intervention of toxicity. Concomitant therapies, including chemotherapy, antibiotics, antifungals, and steroids, may influence the toxicity profile of peg‐asparaginase	100
15	Existing medical conditions may contraindicate the use of peg asparaginase, including active DIC, active DVT, relevant bleeding, CNS thrombosis/bleeding, pre‐ existing pancreatitis, severe pancreatitis with prior asparaginase, and previous severe liver toxicity or disease.	100
16	Although hypersensitivity reactions to asparaginase are not as common in adults as in children. However, hydrocortisone with or without antihistamine and acetaminophen prior to each peg‐asparaginase dose could reduce the incidence of grade 3 and 4 hypersensitivity.	100
17	Although the development of silent inactivation due to neutralizing antibodies may not be clinically evident, searching for anti‐asparaginase antibodies is of questionable value and should not be routinely performed outside research studies in adult ALL.	100
18	In the case of experiencing a grade 3 or 4 hypersensitivity reaction, switching peg‐asparaginase to Erwinia l‐asparaginase is recommended if available.	100
19	Hepatotoxicity (grade 3–4 hyperbilirubinemia) requires close monitoring throughout all therapy phases due to its high frequency. Monitoring may include serum aspartate aminotransferase, alanine aminotransferase, total bilirubin, and direct bilirubin at baseline and weekly for at least 4 weeks after each peg‐asparaginase dose. Liver imaging with ultrasonography before peg‐asparaginase is advisable if the baseline LFT is abnormal or the BMI is above 30.	91
20	To avoid hepatotoxicity, a preventive dose reduction of peg‐asparaginase is advisable in predisposing factors such as chronic liver diseases and BMI > 30, correlating them to patients' age and pre‐existing hepatic steatosis.	100
21	Development of grade 3–4 hepatotoxicity does not contraindicate subsequent administrations of peg‐asparaginase in case of recovery to grade 1 without dose changes.	100
22	Laboratory alterations of the coagulation profile in the absence of clinical signs of thrombosis or bleeding do not necessitate discontinuation of peg‐asparaginase. However, prophylactic anticoagulation with LMWH is advisable.	82
23	Adequate monitoring of fibrinogen is preferred after the initial dose of peg‐asparaginase. However, in the absence of any hemorrhagic symptoms, administering cryoprecipitate or fresh plasma is not indicated.	91
24	Suggested for managing hypertriglyceridemia induced by peg‐asparaginase may include short‐term fasting, a low‐fat diet, oral fibrates, and omega‐3. Insulin/heparin infusion and/or plasmapheresis could be used in more severe cases.	100
25	Any concomitant oral contraceptives or hormone replacement therapy should be discontinued with peg‐asparaginase regimens.	100
26	Osteonecrosis may occur due to asparaginase toxicity and/or the pharmacokinetic interaction of asparaginase with steroids. Monitoring calcium, vitamin D, bone density, and cautious use of steroids are recommended.	100
27	It is recommended to monitor amylase twice weekly at baseline, and twice weekly for 2 weeks after each peg‐asparaginase dose.	82
28	Peg‐asparaginase should be discontinued if grade 3–4 or symptomatic grade 2 pancreatitis develops. Reducing the dose of peg‐ asparaginase should be considered if pancreatitis develops. However, grade 2 pancreatitis does not contraindicate subsequent administration of peg‐asparaginase once resolved.	91

Abbreviations: CNS, central nervous system; DIC, disseminated intravascular coagulopathy; DVT, deep venous thrombosis; hyper‐CVAD, hyperfractionated cyclophosphamide, vincristine, doxorubicin, and dexamethasone; LMWH, low molecular weight heparin.

As previously mentioned, the cumulative body of evidence suggests better survival outcomes with pediatric or pediatric‐inspired regimens than adult regimens for adults with ALL.^11^ Asparaginase is a key component of pediatric ALL regimens, and patients usually receive multiple doses of asparaginase in the post‐remission period. Several randomized controlled trials demonstrated better survival outcomes in patients receiving post‐remission asparaginase than in patients who did not.^40^ However, asparaginase leads to depletion of serum asparagine and the development of a broad spectrum of toxicities, including hepatotoxicity, pancreatitis, and thrombosis.^41^ The panel stated that the frequency of asparaginase‐related toxicities is higher in the pediatric‐inspired regimen (*statement 12*). However, they agreed that pegylated (peg)‐asparaginase toxicities are manageable with dose adjustment in most adult ALL patients and that the life‐threatening complications are rare (*statement 13*). Many centers administered peg‐asparaginase at 1000–2000 IU/m^2^ (compared to 2500 IU/m^2^),^42^, ^43^ exhibiting the same anti‐tumor activities and a more tolerable safety profile. In the UKALL14, the second dose of 1000 IU/m^2^ peg‐asparaginase was omitted to reduce the induction mortality rate.^44^

Previous reports suggested oncologists treating adults with ALL are not familiar with pediatric regimens and related toxicity, which may lead to limited use or premature discontinuation of peg‐asparaginase due to adverse events and limited clinical benefits.^41^ Therefore, consensuses were published to guide oncologists about the prevention and early management of peg‐asparaginase‐associated toxicities.^45^ Several risk factors are incorporated in the development of peg‐asparaginase‐associated toxicities, including older age, obesity, high and multiple doses, several polymorphisms, hypoalbuminemia, thrombocytopenia, history of pancreatitis and liver diseases, history of bleeding or thrombosis, and concurrent steroids.^45^ Thus, oncologists should be aware of the mechanisms of peg‐asparaginase toxicities and at‐risk populations. The experts agreed that risk assessment of the adult patients receiving peg‐asparaginase is crucial to achieving adequate prevention and early management of associated toxicities; particularly, concomitant therapies—such as chemotherapy and steroids‐ may affect the risk of peg‐asparaginase‐associated toxicities (*statement 14*), andthoroughly evaluation for existing medical conditions should be done (*statement 15*).

Asparaginase enzyme can trigger an allergic reaction up to anaphylaxis as it is derived from bacteria; previous reports showed that the overall rate of allergic reaction in adult ALL patients receiving peg‐asparaginase ranges from 7% to 22%, with up to 10% of the patients having grade ≥3.^46^, ^47^ The panel agreed that allergic reaction is less frequent in adults than in children receiving peg‐asparaginase; nonetheless, this disparity in hypersensitivity frequency may be due to the use of prophylactic hydrocortisone rather than age‐specific difference.^41^ Thus, the panel recommended prophylactic hydrocortisone to reduce the risk of grade ≥3 hypersensitivity (*statement 16*). The immune response to peg‐asparaginase typically occurs during the second or third dose and may induce antibody formation leading to treatment failure.^48^ However, the experts agreed that routine measurement of neutralizing antibodies in patients with a history of asparaginase‐induced hypersensitivity has no clinical value (*statement 17*).

Besides, *E.coli* derived asparaginase can be safely replaced by Erwinia l‐asparaginase in cases with grade ≥3 hypersensitivity *(statement 18*). In a compassionate‐use study, patients with a history of grade ≥2 hypersensitivity on *E. coli*–derived asparaginase was switched to Erwinia l‐asparaginase until planned treatment was completed. Nearly 86% of the patients did not experience hypersensitivity, and more than two‐thirds completed their planned asparaginase treatment.^49^

In adult ALL patients, hepatotoxicity is the most frequently observed adverse event during asparaginase cycles; previous reports showed that grade 3/4 hyperbilirubinemia and transaminitis occur in up to 40%–50% of adults on peg‐asparaginase.^46^ Older age, obesity, hypoalbuminemia, thrombocytopenia, and rs4880 polymorphism are among the risk factors for grade 3/4 hyperbilirubinemia in patients receiving peg‐asparaginase. While the exact etiology of hyperbilirubinemia is unclear, it appears within a median of 14 days from induction and rarely leads to clinical symptoms or liver cell failure. Besides, less than 20% of the patients with hyperbilirubinemia develop another episode after re‐challenging.^50^ Although hyperbilirubinemia has no associated clinical symptoms, it can delay subsequent cycles and affect treatment outcomes.^43^ Besides, oncologists with limited experience with peg‐asparaginase may unnecessarily discontinue treatment after hepatoxicity.^41^ Thus, the panel recommended close monitoring of liver function, which should include liver enzymes and bilirubin levels, on a weekly basis for 4 weeks after initiating the peg‐asparaginase. Baseline liver ultrasounds should also be requested in obese patients and patients with impaired liver functions (*statement 19*). At‐risk patients should receive a reduced dose of peg‐asparaginase; however, high‐grade hepatoxicity is not an indication for discontinuing peg‐asparaginase (*statements 20 and 21*).

Asparaginase‐induced thrombosis occurs in 5%–27% of the adult ALL patients,^51^, ^52^ with a higher rate in patients on l‐asparaginase (34%).^53^ The thrombosis usually occurs during the induction phase and is usually venous. ALL is typically associated with a hypercoagulable state, and patients on asparaginase develop thrombosis secondary to low levels of anti‐thrombin, protein C, and plasminogen, among other natural anticoagulants.^54^ Adult ALL patients with older age, obesity, and mediastinal mass at diagnosis are at higher risk of asparaginase‐induced thrombosis.^46^ Therefore, the panel recommended adequate monitoring of the coagulation profile and fibrinogen level after initiating peg‐asparaginase, despite that the current literature indicates that hypofibrinogenemia is associated with a low risk of bleeding in adult ALL patients receiving peg‐asparaginase.^55^ Thus, the experts agreed that an impaired coagulation profile or fibrinogen should not prompt discontinuation of peg‐asparaginase in the absence of clinical signs of thrombosis or bleeding (*statements 22 and 23*). The panel also recommended prophylactic low‐molecular‐weight heparin for high‐risk patients. Recent experience showed a lower rate of asparaginase‐induced thrombosis in adults receiving pediatric regimens with the use of prophylactic anti‐coagulants.^52^

Hypertriglyceridemia is another frequent reversible finding in patients treated with peg‐asparaginase. As with other common asparaginase‐related toxicities, hypertriglyceridemia usually occurs during the first induction cycle, and it is more frequent in obese and older patients.^56^ While hypertriglyceridemia has no clinical manifestations in adult ALL patients, oncologists may reduce the dose or discontinue asparaginase due to the possible association between hypertriglyceridemia and pancreatitis. However, previous reports showed no association between hypertriglyceridemia and pancreatitis in asparaginase‐treated patients.^57^ The GCC experts recommended lifestyle and dietary modifications to manage hypertriglyceridemia induced by peg‐asparaginase (statement 24). Besides, they agreed that oral contraceptives or hormone replacement therapy should be discontinued before initiating peg‐asparaginase (*statement 25*).

Pancreatitis is an infrequent adverse effect of asparaginase that can significantly increase the risk of mortality and morbidities in adult ALL patients; previous reports showed that 5–14% of the adult patients on asparaginase develop pancreatitis, and a higher number of patients showed asymptomatic elevation in pancreatic enzymes.^46^ While the exact etiology of asparaginase‐induced pancreatitis is still unclear, previous reports showed a higher risk of pancreatitis in the high‐risk group and peg‐asparaginase formulation.^58^ In patients with a history of asparaginase‐induced pancreatitis, the rate of recurrence after re‐challenging doses is 46%–63%, with a majority of the cases developing a severe form of the disease.^59^ Thus, current guidelines recommend the discontinuation of peg‐asparaginase and do not advise switching to Erwinia‐derived asparaginase. However, elevated pancreatic enzymes alone are not an indication to stop peg‐asparaginase.^41^ The experts recommended monitoring serum amylase twice weekly for 2 weeks after each peg‐asparaginase dose. Besides, they stated that peg‐asparaginase should be discontinued if grade 3–4 or symptomatic grade 2 pancreatitis develops (*statements 27 and 28*).

### 3.3 Recommendations for future directions and patient‐centered communication

Despite the growing burden of leukemia in GCC countries, the published literature on the treatment and outcomes of adult ALL is scarce. As previously mentioned, the aim of this consensus was to address pediatric‐inspired protocol in comparison to adult protocol for effective treatment of adults' patients. The experts also discussed the need to have extensive meetings and develop regional guidelines covering all aspects of ALL adult management. Also, they recommended future real‐world studies to investigate whether clinical trial results translate into an improvement in survival outcomes in the broader population of adult ALL patients based on risk stratification including age, type of ALL, comorbidities and other factors, in addition, across different ethnicities and geographic backgrounds. Besides, future research should investigate the safety and effectiveness of adjusted doses of peg‐asparaginase for adult ALL. In order to optimize the management landscape of adult ALL, the experts emphasized on the importance of standardizing treatment protocols and guidelines in the GCC region and the establishment of multidisciplinary ALL teams in GCC oncology centers. The GCC oncology centers should also be well‐equipped with advanced diagnostic tools for appropriate risk stratification and follow‐up of the patients. Furthermore, advanced management and up‐to‐date treatments need to be further addressed and updated, such allogeneic HSCT, CAR‐T, and the local infrastructure challenges and regulations. This should be coupled with increased educational awareness and the growing role of pediatric regimens and their associated toxicities among general oncologists; these educational efforts should also include patients and their caregivers to ensure adherence to treatment regimens. Finally, national and regional patient registries are needed to evaluate the long‐term outcomes of adult ALL. (Table 3).

**TABLE 3:** GCC experts' consensus statements on the recommendations for future directions and patient‐centered communication.

No.	Statement	%
29	Population‐based real‐world evidence in the GCC is essential to ensure that clinical trial results are translating into improvements in ALL outcomes across different ethnicities and geographic variances.	100
30	More evidence is required regarding the usage of tailored doses of peg‐asparaginase and/or capping the dose of peg‐asparaginase to test their tolerability and toxicity incidence.	100
31	Multidisciplinary ALL teams are recommended in all GCC oncology therapy sites to include hematologists, clinical nurses, clinical pharmacists, lab specialists, and other relevant specialties.	100
32	Increasing educational awareness regarding disease area knowledge and expected treatment regimens' efficacy and safety among patients & patients' caregivers is essential through various tools including digital platforms and tele‐education.	100
33	There is a need for national/regional ALL‐specialized patient registries to reflect the long‐term follow and outcomes of administered therapies	100
34	There is a need for standardizing a unified protocol for managing adult ALL patients in each center in the GCC to maximize the hands‐on experience on such protocol implementation and the related toxicity management	100
35	All patients should be managed in specialized centers that are well‐equipped and ready to manage all patients.	100

Abbreviations: ALL, acute lymphoblastic leukemia; GCC, Gulf Cooperation Council.

## 4 CONCLUSION

In conclusion, as the management landscape of adult ALL in the GCC is evolving, however, a gap in the management of adult ALL was identified. GCC experts highlighted better response and survival outcomes with pediatric or pediatric‐inspired regiments, especially among AYAs. The use of peg‐asparaginase is a key component of pediatric regimens and has a unique safety profile and manageable adverse effects; even though some general oncologists are less familiar with their usage and handling related toxicities. In this consensus, GCC experts provided a set of recommendations and action plans to optimize the management landscape of adult ALL including, standardized treatment protocols and establishing well‐trained multidisciplinary ALL teams who are well‐equipped with advanced diagnostic tools for appropriate risk stratification and treatment response. Additionally, monitoring and management of peg‐asparaginase‐related toxicities recommendations were also endorsed. Hence, this consensus serves as a potential guideline for ALL management and monitoring of for AYA and adult patients the GCC.

## AUTHOR CONTRIBUTIONS

All authors have given equal contribution for the development of the manuscript. They all have participated in the meeting and shared their experience and scientific input. All authors have revised and approved the final version of this manuscript.

## CONFLICT OF INTEREST STATEMENT

The authors declare that they have no competing interests.

## ETHICS STATEMENT

All experts have orally consented to take part in this consensus development.

## Data Availability

Data available on request from the authors.

## References

[1] Malard F , Mohty M . Acute lymphoblastic leukaemia. Lancet. 2020;395:1146‐1162. doi:10.1016/S0140-6736(19)33018-1 32247396

[2] Yi M , Zhou L , Li A , Luo S , Wu K . Global burden and trend of acute lymphoblastic leukemia from 1990 to 2017. Aging. 2020;12:22869. doi:10.18632/AGING.103982 33203796 PMC7746341

[3] American Cancer Society . Key statistics for acute lymphocytic leukemia (ALL). Am Cancer Soc. 2021;5‐6. https://www.cancer.org/cancer/types/acute-lymphocytic-leukemia/about/key-statistics.html

[4] Howlader N , Ries LA , Krapcho M , et al. SEER cancer statistics review. J Natl Cancer Inst. 2019;111(12):2000‐2016.

[5] Pulte D , Gondos A , Brenner H . Improvement in survival in younger patients with acute lymphoblastic leukemia from the 1980s to the early 21st century. Blood. 2009;113:1408‐1411. doi:10.1182/blood-2008-06-164863 18974371

[6] Siegel RL , Miller KD , Fuchs HE , Jemal A . Cancer statistics, 2022. CA Cancer J Clin. 2022;72:7‐33. doi:10.3322/CAAC.21708 35020204

[7] Sas V , Moisoiu V , Teodorescu P , et al. Approach to the adult acute lymphoblastic leukemia patient. J Clin Med. 2019;8(8):1175‐1203. doi:10.3390/JCM8081175 PMC6722778 31390838

[8] Brown PA , Wieduwilt M , Logan A , et al. NCCN guidelines insights: acute lymphoblastic leukemia, Version 1.2019: featured updates to the NCCN guidelines. J Natl Compr Cancer Netw. 2019;17:414‐423. doi:10.6004/JNCCN.2019.0024 31085755

[9] Larson RA . Managing CNS disease in adults with acute lymphoblastic leukemia. Leuk Lymphoma. 2018;59:3‐13. doi:10.1080/10428194.2017.1326597 28535095

[10] Wieduwilt MJ . How should we treat older adults with Ph+ adult ALL and what novel approaches are being investigated? Best Pract Res Clin Haematol. 2017;30:201‐211. doi:10.1016/J.BEHA.2017.07.001 29050693

[11] Stock W , Luger SM , Advani AS , et al. A pediatric regimen for older adolescents and young adults with acute lymphoblastic leukemia: results of CALGB 10403. Blood. 2019;4(133):1548‐1559. doi:10.1182/BLOOD-2018-10-881961 PMC6450431 30658992

[12] Stock W , La M , Sanford B , et al. What determines the outcomes for adolescents and young adults with acute lymphoblastic leukemia treated on cooperative group protocols? A comparison of Children's Cancer Group and Cancer and Leukemia Group B studies. Blood. 2008;112:1646‐1654. doi:10.1182/BLOOD-2008-01-130237 18502832 PMC2518876

[13] Orellana‐Noia VM , Douvas MG . Recent developments in adolescent and young adult (AYA) acute lymphoblastic leukemia. Curr Hematol Malig Rep. 2018;13:100‐108. doi:10.1007/S11899-018-0442-1 29442287

[14] Gokbuget N , Hoelzer D , Arnold R , et al. Treatment of adult all according to protocols of the German multicenter study Group for Adult all (GMALL). Hematol Oncol Clin North Am. 2000;1(14):1307‐1325. doi:10.1016/S0889-8588(05)70188-X 11147225

[15] Al‐Shahrani ZS , Al‐Rawaji AI , Al‐Madouj AN , Hayder MS . Saudi cancer registry: cancer incidence report Saudi Arabia 2014. Saudi Heal Council; 2017:1‐82.

[16] Bawazir A , Al‐Zamel N , Amen A , Akiel MA , Alhawiti NM , Alshehri A . The burden of leukemia in the Kingdom of Saudi Arabia: 15 years period (1999–2013). BMC Cancer. 2019;19:1‐10. doi:10.1186/S12885-019-5897-5/FIGURES/5 31315607 PMC6637507

[17] Hassan IB , Islam SIAM , Alizadeh H , et al. Acute leukemia among the adult population of United Arab Emirates: an epidemiological study. Leuk Lymphoma. 2009;50:1138‐1147. doi:10.1080/10428190902919184 19557635

[18] Alshemmari SH , Almazyad M , Ram M , John LM , Alhuraiji A . Epidemiology of de novo acute myeloid leukemia in Kuwait per the 2016 WHO Classification. Med Princ Pract. 2022;31:284. doi:10.1159/000524641 35468599 PMC9275004

[19] Alhuraiji A , Abdullah J , Almutairi BO , Albarrak J . General oncology care in Kuwait. In: Al‐Shamsi HO, Abu‐Gheida IH, Iqbal F, Al‐Awadhi A , eds. Cancer in the Arab World. Springer; 2022. doi:10.1007/978-981-16-7945-2_7

[20] Wright JG , Swiontkowski MF , Heckman JD . Introducing levels of evidence to the journal. J Bone Jt Surg: Ser A. 2003;85:1‐3. doi:10.2106/00004623-200301000-00001 12533564

[21] Lynn MR . Determination and quantification of content validity. Nurs Res. 1986;35:382‐386. doi:10.1097/00006199-198611000-00017 3640358

[22] Richard‐Carpentier G , Kantarjian H , Jabbour E . Recent advances in adult acute lymphoblastic leukemia. Curr Hematol Malig Rep. 2019;14(2):106‐118. doi:10.1007/s11899-019-00503-1 30879177

[23] Moorman AV , Schwab C , Ensor HM , et al. IGH@ translocations, CRLF2 deregulation, and microdeletions in adolescents and adults with acute lymphoblastic leukemia. J Clin Oncol. 2012;30:3100‐3108. doi:10.1200/JCO.2011.40.3907 22851563

[24] Marks DI , Paietta EM , Moorman AV , et al. T‐cell acute lymphoblastic leukemia in adults: clinical features, immunophenotype, cytogenetics, and outcome from the large randomized prospective trial (UKALL XII/ECOG 2993). Blood. 2009;114:5136‐5145. doi:10.1182/blood-2009-08-231217 19828704 PMC2792210

[25] Bruneau J , Molina TJ . WHO Classification of Tumors of Hematopoietic and Lymphoid Tissues. Internationaly Agency of Research on Cancer, World Health Organization; 2020:501‐505. doi:10.1007/978-3-319-95309-0_3817

[26] Thomas DA , O'Brien S , Jorgensen JL , et al. Prognostic significance of CD20 expression in adults with de novo precursor B‐lineage acute lymphoblastic leukemia. Blood. 2009;113:6330‐6337. doi:10.1182/blood-2008-04-151860 18703706 PMC2943753

[27] Maury S , Chevret S , Thomas X , et al. Rituximab in B‐lineage adult acute lymphoblastic leukemia. N Engl J Med. 2016;375:1044‐1053. 27626518 10.1056/NEJMoa1605085

[28] Coustan‐Smith E , Mullighan CG , Onciu M , et al. Early T‐cell precursor leukaemia: a subtype of very high‐risk acute lymphoblastic leukaemia. Lancet Oncol. 2009;10:147‐156. doi:10.1016/S1470-2045(08)70314-0 19147408 PMC2840241

[29] Muffly L , Lichtensztajn D , Shiraz P , et al. Adoption of pediatric‐inspired acute lymphoblastic leukemia regimens by adult oncologists treating adolescents and young adults: A population‐based study. Cancer. 2017;123:122‐130. doi:10.1002/CNCR.30322 27622953 PMC5161602

[30] Toft N , Birgens H , Abrahamsson J , et al. Results of NOPHO ALL2008 treatment for patients aged 1–45 years with acute lymphoblastic leukemia. Leukemia. 2018;32:606‐615. doi:10.1038/LEU.2017.265 28819280

[31] DeAngelo DJ , Stevenson KE , Dahlberg SE , et al. Long‐term outcome of a pediatric‐inspired regimen used for adults aged 18–50 years with newly diagnosed acute lymphoblastic leukemia. Leukemia. 2015;29:526‐534. doi:10.1038/LEU.2014.229 25079173 PMC4360211

[32] Ramanujachar R , Richards S , Hann I , et al. Adolescents with acute lymphoblastic leukaemia: outcome on UK national paediatric (ALL97) and adult (UKALLXII/E2993) trials. Pediatr Blood Cancer. 2007;48:254‐261. doi:10.1002/PBC.20749 16421910

[33] Storring JM , Minden MD , Kao S , et al. Treatment of adults with BCR‐ABL negative acute lymphoblastic leukaemia with a modified paediatric regimen. Br J Haematol. 2009;146:76‐85. doi:10.1111/J.1365-2141.2009.07712.X 19438471

[34] Hayakawa F , Sakura T , Yujiri T , et al. Markedly improved outcomes and acceptable toxicity in adolescents and young adults with acute lymphoblastic leukemia following treatment with a pediatric protocol: a phase II study by the Japan Adult Leukemia Study Group. Blood Cancer J. 2014;4:e252. doi:10.1038/BCJ.2014.72 25325302 PMC4220650

[35] Goekbuget N , Stelljes M , Viardot A , et al. First results of the risk‐adapted, MRD‐stratified GMALL trial 08/2013 in 705 adults with newly diagnosed acute lymphoblastic leukemia/lymphoma (ALL/LBL). Blood, 2021;138:362. doi:10.1182/BLOOD-2021-146306

[36] Geyer MB , Ritchie EK , Rao AV , et al. Pediatric‐inspired chemotherapy incorporating pegaspargase is safe and results in high rates of minimal residual disease negativity in adults up to the age of 60 years with Philadelphia chromosome‐negative acute lymphoblastic leukemia. Haematologica. 2021;106:2086. doi:10.3324/HAEMATOL.2020.251686 33054114 PMC8327717

[37] Gökbuget N , Kneba M , Raff T , et al. Adult patients with acute lymphoblastic leukemia and molecular failure display a poor prognosis and are candidates for stem cell transplantation and targeted therapies. Blood. 2012;120:1868‐1876. doi:10.1182/BLOOD-2011-09-377713 22442346

[38] Berry DA , Zhou S , Higley H , et al. Association of minimal residual disease with clinical outcome in pediatric and adult acute lymphoblastic leukemia: A meta‐analysis. JAMA Oncol. 2017;3:e170580. doi:10.1001/JAMAONCOL.2017.0580 PMC5824235 28494052

[39] Samra B , Jabbour E , Ravandi F , Kantarjian H , Short NJ . Evolving therapy of adult acute lymphoblastic leukemia: state‐of‐the‐art treatment and future directions. J Hematol Oncol. 2020;13:17‐70. doi:10.1186/S13045-020-00905-2 32503572 PMC7275444

[40] Pieters R , Hunger SP , Boos J , et al. L‐asparaginase treatment in acute lymphoblastic leukemia: a focus on Erwinia asparaginase. Cancer. 2011;117:238. doi:10.1002/CNCR.25489 20824725 PMC3000881

[41] Aldoss I , Douer D . How I treat the toxicities of pegasparaginase in adults with acute lymphoblastic leukemia. Blood. 2020;26(135):987‐995. doi:10.1182/BLOOD.2019002477 31977001

[42] DeAngelo DJ , Stevenson K , Neuberg DS , et al. A multicenter phase II study using a dose intensified pegylated‐asparaginase pediatric regimen in adults with untreated acute lymphoblastic leukemia: A DFCI ALL Consortium Trial. Blood. 2015;126:80. doi:10.1182/BLOOD.V126.23.80.80 25838348

[43] Goekbuget N , Baumann A , Beck J , et al. PEG‐asparaginase intensification in adult acute lymphoblastic leukemia (ALL): significant improvement of outcome with moderate increase of liver toxicity in the German Multicenter Study Group for Adult ALL (GMALL) Study 07/2003. Blood. 2010;116:494. doi:10.1182/BLOOD.V116.21.494.494

[44] Patel B , Kirkwood AA , Dey A , et al. Pegylated‐asparaginase during induction therapy for adult acute lymphoblastic leukaemia: toxicity data from the UKALL14 trial. Leukemia. 2017;31:58‐64. doi:10.1038/LEU.2016.219 27480385 PMC5154375

[45] Stock W , Douer D , Deangelo DJ , et al. Prevention and management of asparaginase/pegasparaginase‐associated toxicities in adults and older adolescents: recommendations of an expert panel. Leuk Lymphoma. 2011;52:2237‐2253. doi:10.3109/10428194.2011.596963 21827361

[46] Aldoss I , Douer D , Behrendt CE , et al. Toxicity profile of repeated doses of PEG‐asparaginase incorporated into a pediatric‐type regimen for adult acute lymphoblastic leukemia. Eur J Haematol. 2016;96:375‐380. doi:10.1111/EJH.12600 26095294 PMC9496150

[47] Douer D , Aldoss I , Lunning MA , et al. Pharmacokinetics‐based integration of multiple doses of intravenous pegaspargase in a pediatric regimen for adults with newly diagnosed acute lymphoblastic leukemia. J Clin Oncol. 2014;32:905‐911. doi:10.1200/JCO.2013.50.2708 24516026

[48] Liu Y , Smith CA , Panetta JC , et al. Antibodies predict pegaspargase allergic reactions and failure of rechallenge. J Clin Oncol. 2019;37:2051‐2061. doi:10.1200/JCO.18.02439 31188727 PMC6804844

[49] Plourde PV , Jeha S , Hijiya N , et al. Safety profile of asparaginase Erwinia chrysanthemi in a large compassionate‐use trial. Pediatr Blood Cancer. 2014;61:1232‐1238. doi:10.1002/PBC.24938 24436152

[50] Burke PW , Aldoss I , Lunning MA , et al. Pegaspargase‐related high‐grade hepatotoxicity in a pediatric‐inspired adult acute lymphoblastic leukemia regimen does not predict recurrent hepatotoxicity with subsequent doses. Leuk Res. 2018;66:49‐56. doi:10.1016/j.leukres.2017.12.013 29407583

[51] Rytting ME , Thomas DA , O'Brien SM , et al. Augmented Berlin‐Frankfurt‐Münster therapy in adolescents and young adults (AYAs) with acute lymphoblastic leukemia (ALL). Cancer. 2014;120:3660‐3668. doi:10.1002/CNCR.28930 25042398 PMC4239168

[52] Grace RF , DeAngelo DJ , Stevenson KE , et al. The use of prophylactic anticoagulation during induction and consolidation chemotherapy in adults with acute lymphoblastic leukemia. J Thromb Thrombolysis. 2018;45:306‐314. doi:10.1007/s11239-017-1597-7 29260426

[53] Grace RF , Dahlberg SE , Neuberg D , et al. The frequency and management of asparaginase‐related thrombosis in paediatric and adult patients with acute lymphoblastic leukaemia treated on Dana‐Farber Cancer Institute consortium protocols. Br J Haematol. 2011;152:452‐459. doi:10.1111/j.1365-2141.2010.08524.x 21210774 PMC5763913

[54] Payne JH , Vora AJ . Thrombosis and acute lymphoblastic leukaemia. Br J Haematol. 2007;138:430‐445. doi:10.1111/j.1365-2141.2007.06677.x 17608766

[55] Orvain C , Balsat M , Lhéritier V , et al. Prevention of venous thrombotic events in adult patients with acute lymphoblastic leukemia treated in a pediatric‐inspired protocol: a Graall study. Blood. 2016;128:2776. doi:10.1182/blood.v128.22.2776.2776

[56] Persson L , Harila‐Saari A , Hed Myrberg I , Heyman M , Nilsson A , Ranta S . Hypertriglyceridemia during asparaginase treatment in children with acute lymphoblastic leukemia correlates with antithrombin activity in adolescents. Pediatr Blood Cancer. 2017;64:64. doi:10.1002/pbc.26559 28440015

[57] Raja RA , Schmiegelow K , Sørensen DN , Frandsen TL . Asparaginase‐associated pancreatitis is not predicted by hypertriglyceridemia or pancreatic enzyme levels in children with acute lymphoblastic leukemia. Pediatr Blood Cancer. 2017;64:32‐38. doi:10.1002/pbc.26183 27555294

[58] Oparaji JA , Rose F , Okafor D , et al. Risk factors for Asparaginase‐associated pancreatitis: a systematic review. J Clin Gastroenterol. 2017;51:907‐913. doi:10.1097/MCG.0000000000000827 28375864 PMC11488675

[59] Wolthers BO , Frandsen TL , Baruchel A , et al. Asparaginase‐associated pancreatitis in childhood acute lymphoblastic leukaemia: an observational Ponte di Legno Toxicity Working Group study. Lancet Oncol. 2017;18:1238‐1248. doi:10.1016/S1470-2045(17)30424-2 28736188

